# Acute kidney injury does not explain sex differences in kidney replacement therapy initiation or death amongst individuals with chronic kidney disease reported to the UK Renal Registry

**DOI:** 10.1093/ckj/sfaf105

**Published:** 2025-04-18

**Authors:** Takahiro Tsuji, Anna Casula, Laurie Tomlinson, Dorothea Nitsch, Barnaby Hole

**Affiliations:** Department of Non-Communicable Disease Epidemiology, London School of Hygiene and Tropical Medicine, London, UK; UK Renal Registry, UK Kidney Association, Bristol, UK; Department of Non-Communicable Disease Epidemiology, London School of Hygiene and Tropical Medicine, London, UK; Department of Non-Communicable Disease Epidemiology, London School of Hygiene and Tropical Medicine, London, UK; UK Renal Registry, UK Kidney Association, Bristol, UK; Renal Unit, Royal Free London NHS Foundation Trust, London, UK; UK Renal Registry, UK Kidney Association, Bristol, UK; Population Health, University of Bristol, Bristol, UK

**Keywords:** acute kidney injury, chronic kidney disease, kidney replacement therapy, mortality, sex

## Abstract

**Background:**

Why more males than females start kidney replacement therapy (KRT) is incompletely understood. Acute kidney injury (AKI) is a possible factor underlying sex differences in chronic kidney disease (CKD) progression, but previous studies regarding this have been inconclusive. We investigated sex differences in the association between AKI and CKD progression in UK nephrology care.

**Methods:**

This cohort study uses UK Renal Registry data. Adults with CKD stages 4/5 in 14 nephrology centres in England were followed from January 2018 to December 2021. We compared their baseline characteristics by sex and calculated cause specific hazard ratio (HR) for outcomes: time to AKI stage 2/3 (AKI2/3), initiation of chronic KRT and death by all causes.

**Results:**

A total of 15 547 patients were included. Fewer females (43.8%) were seen in renal centres than males (56.2%). During follow-up, 3909 (25.1%) AKI2/3 episodes, 3510 (22.6%) KRT initiations, and 7293 (46.9%) deaths were observed. Males were more likely than females to experience each outcome: AKI2/3 [adjusted HR 1.39, 95% confidence interval (CI) 1.31–1.49], KRT initiation (adjusted HR 1.51, 95% CI 1.39–1.65) and death (adjusted HR 1.11, 95% CI 1.05–1.16). Adjustment for AKI2/3 did not change the association between being male and the higher risk of KRT initiation.

**Conclusion:**

Being male was associated with a higher risk of AKI2/3, KRT initiation and death. Fewer females appeared in nephrology care data than expected from population CKD prevalence. However, no evidence was found to support the hypothesis that AKI2/3 explains the higher KRT initiation rates seen amongst males.

KEY LEARNING POINTS **What was known:**

- Chronic kidney disease (CKD) is more prevalent in females than in males, whilst more males than females start dialysis and receive kidney transplants.
- Studies have shown inconsistent associations between sex and the risk and severity of acute kidney injury (AKI).
- Whether sex differences in AKI explain why more males than females with CKD progress to kidney replacement therapy (KRT) has not been well studied.

**This study adds:**

- No evidence was found to support AKI2/3 explaining the higher KRT initiation rates seen amongst males with CKD when compared with females.
- Fewer females appeared in UK nephrology CKD care data than expected from population CKD prevalence.
- Males with CKD more frequently experienced AKI stage 2/3, KRT and death than females.

**Potential impact:**

- Fewer females than males access nephrology care. The reasons underlying differential access by sex need explanation.
- Interventions targeted at prevention of adverse renal events following AKI stage 2/3 are likely to offer similar benefits to males and females.

## INTRODUCTION

Chronic kidney disease (CKD) is a significant public health issue globally, resulting in avoidable morbidity and mortality. More than 700 million people have CKD, with approximately 6 million in the UK [1]. Since CKD is a major independent risk factor for cardiovascular disease, only a minority of those will reach kidney failure before death. However, initiation of kidney replacement therapy (KRT—dialysis or kidney transplantation) comes at considerable burden to those who start, and to healthcare systems [2].

More females than males meet creatinine-based diagnostic thresholds for CKD [3]. However, rates of dialysis initiation and kidney transplantation are consistently higher amongst males than females [3–7]. Furthermore, males appear to start KRT younger and at higher estimated glomerular filtration rate (eGFR) than females [8]. This appears consistent across nations, ages and ethnicities [9–12]. Both healthy males [13] and those with CKD [4, 7, 14] appear to experience more rapid decline in GFR than females. However, meta-analysis of individual participant data from 46 general population and CKD cohorts showed no evidence of a sex difference in associations between eGFR and urinary albumin-to-creatinine ratio with kidney failure risk [15]. Numerous potential explanations for the paradoxical pattern in CKD prevalence and progression have been advanced [10], including effects of sex hormones, between-sex differences in risk factors such as diabetes, hypertension, obesity and smoking, uptake of conservative kidney management [16–18], and acute kidney injury (AKI) [19–22].

AKI reflects an heterogenous clinical syndrome, diagnosed when a rapid increase in serum creatinine (SCr) or decline in urine output indicates abrupt onset of organ dysfunction [23]. Studies in various settings have shown inconsistent associations between sex and the risk and severity of AKI [10]. Meta-analyses and nationwide studies have indicated that males are more likely than females to experience AKI and need KRT in the hospital inpatient setting [24–27]. Meanwhile, males appear more likely than females to recover native kidney function following AKI [28–30], and female sex has been identified as a risk factor for progression to advanced CKD [31]. We used data from the UK Renal Registry (UKRR) to examine the hypothesis that sex differences in AKI may explain why more males than females with advanced CKD progress to KRT.

## MATERIALS AND METHODS

### Study design and setting

We conducted a retrospective cohort study of patients receiving National Health Service (NHS) nephrology CKD care in England, UK, using routinely collected UKRR data. Nephrology services in England are required to report individuals with CKD under their care to the UKRR. The UKRR reports on the care of individuals with eGFR <30 mL/min/1.73 m^2^, including patients who have newly started CKD care, and those already receiving treatment [9]. AKI data are generated from an NHS-approved automated algorithm, which draws upon a national network of biochemistry laboratory data to flag individuals with a rapidly rising SCr on inpatient and outpatient laboratory tests [32]. AKI alert data are housed in a ‘Master Patient Index’ (MPI) held by the UKRR. All analyses described in this study were performed using data from a subset of 14 of the 48 (29%) English kidney units. This represents all available relevant data, with units chosen because they had been submitting both CKD data to the UKRR from 2017 to the latest available data in 2021, and the main laboratory covering the kidney unit had been contributing to the MPI during 2018–21. Patients were considered eligible if they had been reported to UKRR as receiving specialist CKD before, and were aged ≥18 years on 31 December 2017. Inclusion criteria required at least one SCr measurement indicating a baseline eGFR <30 mL/min/1.73 m^2^ in 2017 (Chronic Kidney Disease Epidemiology Collaboration 2009 without racial adjustment [33] using the latest SCr in 2017), and no history of chronic KRT before 1 January 2018. Individuals were followed until the earliest of (i) 31 December 2021, (ii) death, (iii) loss to follow-up or (iv) the outcome of interest: index AKI stage 2 and 3 (AKI2/3) or initiation of chronic KRT, depending upon the analysis. To examine the effect of the COVID-19 outbreak, we conducted a sensitivity analysis with follow-up to 31 December 2019.

### Variables and data sources

We extracted individual patient sex (male/female, as per medical record), date of chronic KRT initiation, death by all causes (last day of the month of death), age, ethnicity, Index of Multiple Deprivation (IMD), baseline eGFR, SCr measurement frequency, urine albumin-to-creatinine ratio (uACR), urine protein-to-creatinine ratio (uPCR) and primary kidney disease from UKRR data. Comorbidity data (presence or absence of chronic obstructive pulmonary disease (COPD), cardiovascular disease, diabetes, heart failure, malignancy and peripheral vascular disease) were provided from linked Hospital Episode Statistics admitted patient care, and outpatient care [34, 35]. Data relating to disease severity (for example HbA1c) were unavailable. Age was calculated for the 31 December of 2017 and categorized into four groups with consideration of similar sample size: 18–65 years old; 66–75 years old; 76–85 years old; and ≥86 years old. Ethnicity was grouped as Asian, Black, White and other. Socioeconomic status was categorized by IMD quintile, from least deprived to most deprived. Baseline eGFR was categorized into stage 5 (CKD5: eGFR <15 mL/min/1.73 m^2^) and 4 (CKD4: 15 mL/min/1.73 m^2 ^≤ eGFR < 30 mL/min/1.73 m^2^). SCr measurement frequency was defined as the number of SCr records in 2017. Baseline uACR and uPCR were defined by the latest measurement in 2017. The MPI dataset was used to identify and stage AKI alerts. AKI2/3 was chosen as a proxy for more severe AKI, which remains distinguishable even in the advanced CKD. We identified onset of AKI2/3 where a SCr value reached (i) >1.5× the individual's reference value and >354 μmol/L; (ii) or more than two times higher than the individual's reference value (their lowest SCr if results available within 7 days, or the median SCr if results available within 8–365 days) [32]. We defined AKI2/3 in 2017 and during follow-up separately as ‘baseline AKI2/3’ and ‘AKI2/3 during follow-up’, respectively. AKI2/3 during follow-up was accounted for as a time-varying covariate. At time zero (1 January 2018), AKI status was set to zero, and after the first (index) AKI2/3 alert during follow-up, AKI status adopted the value of one. Accommodation of subsequent AKI2/3 alerts was tested, but this did not improve the models, so was not included in further analyses.

### Statistical methods

The main exposure of interest was sex. We chose three outcomes relating to CKD progression. The primary outcome was time to initiation of chronic KRT, including kidney transplantation and dialysis. The two secondary outcomes were (i) time to AKI2/3 and (ii) time to all-cause death. Variables identified as potential confounders for adjustment were age, ethnicity, IMD, SCr measurement frequency, baseline AKI2/3, AKI2/3 during follow-up and comorbidities. The presence of hypertension was not adjusted for, given the high prevalence in stage 4 and 5 CKD amongst patients of both sexes. Of the possible confounders considered, uACR and uPCR were excluded from the analysis because of the high level of missing data.

We presented descriptive analysis by sex, including clinical and demographic patient features. To look for between-sex differences, we conducted Kruskal–Wallis tests for non-normally numerical variables and Chi-squared tests for categorical variables. Time to event analysis was performed using a Cox-proportional hazard model for the primary analysis of KRT initiation and the two secondary outcomes, AKI2/3 and all-cause death. The results were calculated as cause specific hazard ratios with 95% confidence intervals (95% CI).

For the AKI2/3 event analysis, KRT and death were the competing events. For the KRT event analysis, death was the competing event, while for the death without KRT analysis, KRT was the competing event. To account for competing risk, the cumulative incidence curve was calculated as a cumulative incidence function for AKI2/3, KRT initiation, and death without KRT initiation, while the incidence of death was calculated by the Kaplan–Meier method. For all analyses, censoring was the end of follow-up (December 2021). We carried out sensitivity analyses using Fine and Gray model. We tested for interactions between sex and AKI2/3 at baseline and during follow-up. We set the significance level in the statistical tests as 5% on both sides. We conducted the statistical analysis using SAS9.4 (SAS Institute Inc., Cary, NC, USA). Reporting was based on the Strengthening the Reporting of Observational Studies in Epidemiology (STROBE) statement [36].

### Ethics

The UKRR holds research ethics approval allowing collection of data without personal consent as per the Confidential Advisory Group for research and audit purposes. Institutional ethical approval was obtained from the London School of Hygiene and Tropical Medicine Research Ethics Committee (LSHTM MSc Ethics Ref: 27 042).

## RESULTS

### Characteristics of the cohort

**Figure 1:**
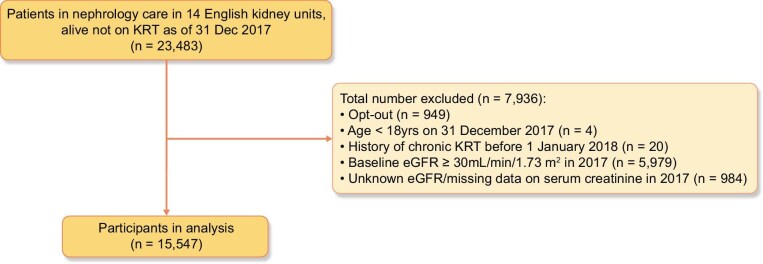
Flow diagram of participants included in analysis KRT - kidney replacement therapy; eGFR - estimated glomerular filtration rate.

The 14 units reported 15 547 individuals as receiving nephrology CKD care to the UKRR at the end of 2017, with last eGFR in 2017 <30 mL/min/1.73 m^2^ (Fig. 1). The proportion of females in the analysis dataset was substantially smaller than males (females, 43.8%; males, 56.2%) (Table 1). Whilst females were older, and the difference reached the alpha 0.05 threshold, the absolute difference was marginal [median age females 78 (IQR 67–84) years, males 77 (IQR 67–84) years, *P *= .017]. Females were more socioeconomically deprived (*P *< .001), had less frequent SCr measurement (*P *= .003) and had a lower proportion of CKD5 (*P *= .002). Females had lower rates of malignancy (*P *< .001) and peripheral vascular disease (*P *< .001). Other comorbidities appeared equally frequently between sexes. Proteinuria data were highly missing—87.3% for uACR and 78.8% for uPCR—so were not included in further analyses.

**Table 1:** Characteristics of participants in the analysis (*n* = 15 547).

	All		Male		Female		
Variables	*N*	%	*N*	%	*N*	%	*P*-value
Total	15 547		8739	56.2	6808	43.8	
Age							
Median (IQR)	77 (67–84)		77 (67–84)		78 (67–84)		.0170
18–65 years	3423	22.0	1930	22.1	1493	21.9	>.0001
66–75 years	3637	23.4	2154	24.6	1483	21.8	
76–85 years	5584	35.9	3077	35.2	2507	36.8	
≥86 years	2903	18.7	1578	18.1	1325	19.5	
IMD							
1—most deprived	2526	16.3	1288	14.7	1238	18.2	>.0001
2	3100	19.9	1656	18.9	1444	21.2	
3	3328	21.4	1892	21.7	1436	21.1	
4	3381	21.8	1976	22.6	1405	20.6	
5—least deprived	3212	20.7	1927	22.1	1285	18.9	
Ethnicity							
White	13 299	85.5	7556	86.5	5743	84.4	>.0001
Asian	1336	8.6	677	7.7	659	9.7	
Black	454	2.9	244	2.8	210	3.1	
Other	324	2.1	184	2.1	140	2.1	
Missing	134	0.9	78	0.9	56	0.8	
Primary renal disease							
Diabetes	2038	13.1	1247	14.3	791	11.6	>.0001
Glomerulonephritis	668	4.3	443	5.1	225	3.3	
Hypertension	832	5.4	494	5.7	338	5.0	
Polycystic kidney disease	473	3.0	247	2.8	226	3.3	
Pyelonephritis	697	4.5	425	4.9	272	4.0	
Renal vascular disease	1076	6.9	665	7.6	411	6.0	
Other	1185	7.6	610	7.0	575	8.4	
Uncertain aetiology	2210	14.2	1137	13.0	1073	15.8	
Missing	6368	41.0	3471	39.7	2897	42.6	
Comoridity							
COPD	2985	19.2	1676	19.2	1309	19.2	.9400
CVD	1536	9.9	879	10.1	657	9.7	.4000
Diabetes	6251	40.2	3552	40.6	2699	39.6	.2100
Heart failure	3266	21.0	1849	21.2	1417	20.8	.6000
Malignancy	2373	15.3	1628	18.6	745	10.9	>.0001
PVD	1345	8.7	938	10.7	407	6.0	>.0001
No. comorbidity							
None	5571	35.8	2988	34.2	2583	37.9	>.0001
1	4818	31.0	2660	30.4	2158	31.7	
≥2	5158	33.2	3091	35.4	2067	30.4	
SCr measurement frequency							
Median (IQR)	3 (2–5)		4 (2–5)		3 (2–5)		.0033
Proteinuria measurement							
Patient with proteinuria data	4281	27.54	2496	28.6	1785	26.2	.0012
Baseline eGFR							
Median (IQR)	20.6 (15.6–24.7)		20.5 (15.3–24.6)		20.6 (15.8–24.8)		.0100
CKD stage							
stage 4	12 009	77.2	6671	76.3	5338	78.4	.0020
stage 5	3538	22.8	2068	23.7	1470	21.6	

CVD, cardiovascular disease; PVD, peripheral vascular disease.

### Outcome analysis

**Figure 2:**
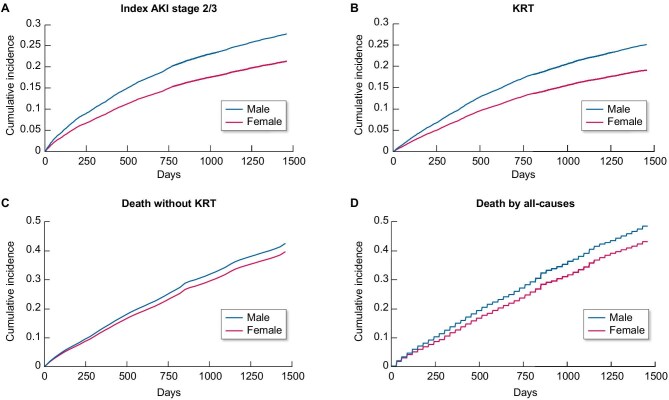
Cumulative incidence of (A) index AKI stage 2/3, (B) KRT, (C) death without KRT, and (D) death by all-causes. A, B, and C were calculated as the cumulative incidence function. D was calculated as (1- Kaplan-Meier function). AKI - acute kidney injury; KRT - kidney replacement therapy.

A total of 46 779 person-years were included in the cohort with an average of 3.0 person-years per individual. Between 1 January 2018 and 31 December 2021, 3909 (25.1%) index AKI2/3 events, 3510 (22.6%) chronic KRT initiations and 7293 (46.9%) deaths were observed. Males were more likely to experience AKI, KRT and death than females (Fig. 2). Per 1000 person-years, more males than females experienced AKI2/3 [females, 85; males, 123; crude hazard ratio (HR) 1.43, 95% CI 1.34–1.53]; initiated chronic KRT (females, 62; males, 85; crude HR 1.43, 95% CI 1.34–1.54); and died (females, 143; males, 167; crude HR 1.17, 95% CI 1.12–1.23). Most of the AKI2/3 during follow-up happened in the first 2 years (Appendix 1).

In the fully adjusted model, males remained more likely than females to develop AKI2/3 (adjusted HR 1.39, 95% CI 1.31–1.49), initiate KRT (adjusted HR 1.51, 95% CI 1.39–1.65) and all-cause death (adjusted HR 1.11, 95% CI 1.05–1.16). Adjustment for baseline AKI2/3 and AKI2/3 during follow-up minimally attenuated the association between being male and higher risk of KRT initiation, and attenuated the association between being male and higher risk of death (Table 2). Death without KRT was more common in males than females in crude and adjusted analysis (crude HR 1.17, 95% CI 1.12–1.23, adjusted HR 1.13, 95% CI 1.06–1.20) (Fig. 2 and Table 2).

**Table 2:** Crude and adjusted cause-specific HR by sex (males to females) for AKI2/3, KRT initiation, death without KRT and death by all causes (*n* = 15 547).

	AKI stage 2/3			KRT initiation			Death without KRT			Death by all-causes		
	HR	95% CI	*P*-value	HR	95% CI	*P*-value	HR	95% CI	*P*-value	HR	95% CI	*P*-value
Model 1: crude model	1.43	(1.34–1.53)	>.0001	1.43	(1.34–1.54)	>.0001	1.17	(1.12–1.23)	>.0001	1.17	(1.12–1.23)	>.0001
Model 2: adjustment without AKI	1.40	(1.31–1.50)	>.0001	1.53	(1.41–1.65)	>.0001	1.19	(1.13–1.26)	>.0001	1.18	(1.13–1.24)	>.0001
Model 3: Model 2 + baseline AKI adjustment	1.39	(1.31–1.49)	>.0001	1.52	(1.41–1.65)	>.0001	1.19	(1.13–1.26)	>.0001	1.18	(1.12–1.23)	0.0001
Model 4: Model 3 + AKI during follow-up adjustment				1.51	(1.39–1.65)	>.0001	1.13	(1.06–1.20)	>.0001	1.11	(1.05–1.16)	>.0001

Model 1: unadjusted crude model; Model 2: adjusted by age groups, IMD, ethnicity, comorbidity, SCr measurement frequency, baseline eGFR;

Model 3: additionally adjusted for baseline AKI2/3 on Model 2; Model 4: additionally adjusted for AKI2/3 during follow-up on Model 3.

Comorbidities were adjusted for each comorbidity individually (COPD, cardiovascular disease, diabetes, heart failure, malignancy and peripheral vascular disease).

### Sensitivity analysis

A sensitivity analysis excluding the post-COVID-19 (from January 2020) period from the follow-up slightly changed the HR seen in the full cohort, but without substantial change in associations seen (Appendix 2). We found no evidence in Fine and Gray models that the competing risk of death explained the differences between males and females with regards to KRT initiation (Appendix 3). We found no evidence of interaction between AKI2/3 at baseline and sex (*P*-value interaction .66), or between AKI2/3 during follow-up and sex (*P*-value interaction .15) (data not shown).

## DISCUSSION

### Key findings and interpretation

To our knowledge, this is the first study to examine the effect of AKI on sex differences in CKD progression in UK nephrology care. We found that being male was associated with higher risk of AKI2/3, KRT initiation and all-cause death. Males were also more likely to experience death without KRT. While we hypothesized that sex differences in AKI2/3 may explain males’ higher risk of KRT initiation, our findings did not support this. Males’ higher risk of KRT initiation and death were only marginally attenuated by adjustment for baseline AKI2/3 and AKI2/3 during follow-up. Our data are consistent with the previous literature. KRT incidence in males being greater than females has been observed in all European countries [7]. In terms of our analysis results, we found very similar estimates in competing risk analyses to those seen previously in Sweden [37].

The proportion of females in our cohort (43.8%) was smaller than that reported in other studies and does not mirror the reported population sex distribution. For example, Hirst *et al*. (2020) reported that the proportion of females in CKD4 patients in UK primary care was 50.7% [38]. A Swedish study found that processes of care for people with CKD were gendered, with lower diagnosis rates in women, and less referral when presenting with advanced CKD when compared with men [39]. A similar phenomenon may be present in UK clinical care, though we do not have data in this study to confirm the smaller proportion of females in nephrology care reflect referral patterns.

Our sensitivity analyses indicated that the associations between male sex and an increased risk of all outcomes was slightly stronger in models that included the COVID-19 period compared with those that excluded it. It is known that CKD patients were at high risk of adverse outcomes during the pandemic [40]. Our data suggest that the impact of COVID-19 may have been more pronounced in males than females receiving nephrology care.

### Strengths and limitations

The strengths of our study lie in the use of a large cohort, drawing upon a national registry, linked secondary care data, and a national AKI alert system. The cohort included multiple UK renal centres, with prospective data collection meaning measurements are largely free from outcome or exposure status. Meanwhile, the population served and practice patterns of the 14 included units may not be representative of UK practice, and findings cannot be assumed to generalize to other countries. Sex was ascertained from medical records as a binary categorical variable, meaning we were unable to investigate effects in transgender, non-binary and gender-diverse individuals.

There are limitations to our study. We did not include proteinuria data in the analysis due to the highly incomplete proteinuria data. Whilst proteinuria is not used to stage acute kidney disease, it is a major risk factor for adverse kidney outcomes [41, 42], is elevated after AKI [43], and differs by sex [10]. The severity of underlying CKD in our cohort may have been misestimated in the absence of proteinuria data, undermining our ability to resolve relationships between AKI and CKD [44], and to spot sex differences [45, 46]. Other important sex differences are also uncaptured in our data. For example, biological factors, such as the higher frequency of bladder outflow obstruction in males [47], or the differential effect of diabetes on disease progression in females [7]. We identified our exposure of AKI2/3 through the NHS AKI alert, which is based on SCr testing, which occurs mainly during hospital admissions. Since AKI alerts are triggered only when tests are performed, we may have missed cases of AKI in female patients, if they experienced fewer admissions. The unavoidable lack of a ‘prior AKI’ variable (predating 2017) is also a significant limitation, given the recurrent nature of AKI in people with CKD, especially since AKI may have triggered referral to specialist services. If females with CKD are referred later or only when their condition is more severe, our sample may include women with a higher disease burden than men. However, we adjusted for comorbidities, so the difference in disease severity should be partly accounted for. Additionally, if referral bias leads to an underrepresentation of females with less severe CKD in nephrology care, this might attenuate the higher risk of AKI2/3, KRT initiation and death in males. Consequently, our results reflect a conservative estimate of the higher risk in males for these outcomes.

Medication information was unavailable, preventing us from examining whether the use of cardio/reno-protective drugs varied by sex. Our prevalent cohort includes people of unknown durations of CKD4 and CKD5 diagnoses prior to referral, which may introduce healthier survivor bias. Since our cohort includes only patients already under nephrology care, it does not represent the entire population of individuals with CKD4 and CKD5. Furthermore, included centres might not be representative, potentially introducing selection bias and limiting the generalizability of our findings—especially if referral practices differ by sex.

Like many other studies, our data show an association between AKI in advanced CKD and higher rates of KRT initiation and mortality. Whilst our data do not support a relationship between AKI episodes and the higher rate of kidney failure in males, our findings do not prove that this relationship does not exist. Whilst we accounted for CKD stage at baseline, it is possible that sex-effects have already been established before individuals reach advanced CKD, requiring analysis in a more ‘upstream’ dataset than our own. Alternatively, longer follow-up than the 4 years of data available to us may be required to capture the full influence of AKI, especially if this tends to happen late in the disease course.

## CONCLUSION

We found that being male was associated with higher risk of AKI2/3, KRT initiation and death in UK nephrology care. Fewer females than males accessed nephrology care. Whilst more males than females accessed nephrology care, and males experienced more AKI2/3 than females, accounting for AKI2/3 did not meaningfully change the males’ higher rate of KRT initiation. Prospective population level studies with ascertainment of proteinuria, and/or linkage between primary and secondary care records, may help to ensure that comparable groups of males and females are used in future studies attempting to explain why more males start KRT than females.

## Data Availability

Data cannot be shared for ethical/privacy reasons. Applications for data are welcomed by the UK Renal Registry, please see UKRR data | The UK Kidney Association.

## Appendix

**Appendix 1:** Proportion of AKI2/3 during the follow up (*n* = 15 547).

Time period during follow-up	No first AKI2/3, *n* (%)	Cumulative proportion (%)
0–0.5 years	1033 (26.4)	26.4
0.5–1 years	612 (15.7)	42.1
1–1.5 years	598 (15.3)	57.4
1.5–2 years	469 (12.0)	69.4
2–2.5 years	384 (9.8)	79.2
2.5–3 years	275 (7.0)	86.2
3–3.5 years	294 (7.5)	93.8
3.5–4 years	244 (6.2)	100.0
Total	3909 (100.0)	

## Appendix

**Appendix 2:** Cause specific HR by sex (males to females) in 2 years’ follow-up and in 4 years’ follow-up.

	AKI stage 2/3			KRT initiation			Death without KRT			Death by all-causes		
	HR	95% CI	*P*-value	HR	95% CI	*P*-value	HR	95% CI	*P*-value	HR	95% CI	*P*-value
2 years follow-up	1.36	(1.25–1.47)	>.0001	1.46	(1.32–1.61)	>.0001	1.09	(1.01–1.18)	.02	1.06	(0.99–1.13)	.09
4 years follow-up	1.39	(1.31–1.49)	>.0001	1.51	(1.39–1.65)	>.0001	1.13	(1.06–1.20)	>.0001	1.11	(1.05–1.16)	>.0001

AKI—acute kidney injury; KRT—kidney replacement therapy; HR—hazard ratio; 95%CI–95% confidence interval

## Appendix

**Appendix 3:** Crude and adjusted subdistribution HR by sex (males to females) for AKI2/3, KRT initiation and death without KRT (*n* = 15 547).

	AKI stage 2/3			KRT initiation			Death without KRT			Death by all-causes		
	HR	95% CI	*P*-value	HR	95% CI	*P*-value	HR	95% CI	*P*-value	HR	95% CI	*P*-value
Model 1: crude model	1.36	(1.27–1.45)	>.0001	1.36	(1.27–1.46)	>.0001	1.10	(1.04–1.15)	>.0001	NA		
Model 2: adjustment without AKI	1.32	(1.24–1.41)	>.0001	1.47	(1.36–1.58)	>.0001	1.12	(1.06–1.18)	>.0001	NA		
Model 3: Model 2 + baseline AKI adjustment	1.32	(1.23–1.41)	>.0001	1.48	(1.37–1.59)	>.0001	1.12	(1.06–1.18)	>.0001	NA		
Model 4: Model 3 + AKI during follow-up adjustment				1.44	(1.32–1.56)	>.0001	1.04	(0.99–1.10)	.134	NA		

Model 1: unadjusted crude model; Model 2: adjusted by age groups, IMD, ethnicity, comorbidity, SCr measurement frequency, baseline eGFR;

Model 3: additionally adjusted for baseline AKI2/3 on Model 2; Model 4: additionally adjusted for AKI2/3 during follow-up on Model 3.

Comorbidities were adjusted for each comorbidity individually (COPD, cardiovascular disease, diabetes, heart failure, malignancy and peripheral vascular disease).
